# Uterine Malignancy: Pathological Pattern and Changing Incidence in a Teaching Hospital in Jeddah, Saudi Arabia

**DOI:** 10.7759/cureus.42074

**Published:** 2023-07-18

**Authors:** Abdulrahim K Turkistani, Layla Abdullah

**Affiliations:** 1 Anatomical Pathology, King Abdulaziz University Hospital (KAUH), Jeddah, SAU

**Keywords:** type 2 endometrioid carcinoma, type 1 endometrioid carcinoma, serous carcinoma, uterine bleeding, endometrial carcinoma, malignancy

## Abstract

Introduction: Uterine carcinoma is one of the most common malignancies affecting women globally. It is the second most common gynecologic malignancy in impoverished countries and the most common in industrialized countries.

Objective: To describe the histopathological patterns of uterine malignancies and their changing incidence at King Abdulaziz University Hospital from 2011 to 2020.

Methodology: A retrospective study was conducted to analyze female patient record files from 2011 to 2020 who underwent uterine resection surgery at the King Abdulaziz University Hospital, Jeddah, Saudi Arabia.

Results: A total of 263 patients were included. The age ranged from 30 to 95 years old; median age of 61 years. The majority (71%) presented with the complaint of abnormal uterine bleeding. The most common histopathological diagnosis was endometrioid carcinoma (70%), followed by serous carcinoma (13.7%). The lowest reported uterine cancer diagnoses were in the year of 2011 with (<5%) of the cases while the highest were in the year of 2020. These findings demonstrate a variable incidence of endometrial carcinomas in the study population over the study period. With trends of an increasing incidence till 2018 followed by a marginal reduction in 2019 and 2020.

Conclusion: The most frequent histopathological diagnosis of uterine cancer was endometrioid carcinoma followed by serous carcinoma, (70%) and (13.7%) respectively. Type 1 endometrial carcinoma was prevalent throughout the decade surpassing the type-2 endometrial carcinoma. The trend also shows a steady increase in the frequency of uterine cancer which is alarming and prompts further research to determine factors associated with and molecular classification of reported uterine cancer cases.

## Introduction

Uterine malignancies are a type of cancer that originates in the tissues of the uterus. Uterine carcinoma is one of the most common tumors that affect women worldwide. The endometrium of the uterus, where 90% of uterine malignancies originate, is where endometrial cancer develops [1]. Due to the large burden of uterine cancer and its associated morbidities and mortalities, it is of great importance to investigate the incidence and emphasize the magnitude of the condition to guide national programs of prevention and control.

## Materials and methods

Study settings

We conducted a retrospective study at King Abdulaziz University Hospital, Jeddah, Saudi Arabia. The data was collected from December 2021 to December 2022 and included cases from 2011 to 2020.

Study population

The study included women who underwent uterine resection surgery at the hospital. Patients aged 30 years or above who were diagnosed with any uterine malignancy were eligible to be included in the study. Patients with incomplete data or those who underwent surgeries in other hospitals were excluded. No specific sample size formula was used; all patients who met the eligibility criteria were included in the study. A total of 263 patients were included.

Data collection

Data were collected retrospectively from the electronic medical records of the included patients. The clinical notes and histopathological reports of each patient were examined to extract data related to the study. The collected variables included age, presenting symptoms, year of diagnosis, radiological findings, histopathological diagnosis, and type of surgery performed.

Statistical analysis

The data were analyzed using IBM SPSS Statistics for Windows, Version 27.0 (IBM Corp., Armonk, NY). The median and interquartile range (IQR) were used for continuous variables after performing the Shapiro-Wilk test of normality. Proportions were used to summarize categorical variables. Chi-square and Fisher's exact tests were used to find significant associations with types of histopathological diagnosis. The significance level was set at 0.05.

Ethical considerations

This study was conducted in accordance with the Declaration of Helsinki. Informed consent was not required as data were collected retrospectively. Patient confidentiality was maintained throughout the study.

## Results

A total of (263) uterine malignancy patients were included in the analysis. The age ranged from 30-95 years with a median of 61, IQR (52-67) years. The most common presenting symptom was abnormal vaginal bleeding (79.5%), followed by abdominal pain (9.5%), and abdominal distention (3.8%) respectively. A total of (7.2%) had other symptoms. About half (45.6%) did not have previous radiological images, (38.8%) had endometrial thickening, (10.6%) showed uterine mass and (4.9%) had other findings. Histopathological diagnosis revealed type 1 as the most common type in 70% of the patients, type 2 among 22%, and other findings among 8%. Details of age categories, complaints, and histopathological diagnosis are presented in (Table 1).

**Table 1:** Clinical presentation, radiological and histopathological characteristics of uterine malignancy among the patients *Serous carcinoma 90%, endometrioid carcinoma 10%

Variable	Groups	N	%
Age category	30-40	26	9.9%
	41-50	36	13.7%
	51-60	69	26.2%
	>60	132	50.2%
Complain	Abnormal vaginal bleeding	209	79.5%
	Abdominal pain	25	9.5%
	Abdominal distention	10	3.8%
	Others	19	7.2%
Radiology	No previous images	120	45.6%
	Endometrial thickening	102	38.8%
	Uterine mass	28	10.6%
	Others	13	4.9%
Diagnosis	Carcinosarcoma	19	7.2%
	Clear cell carcinoma	3	1.1%
	Endometrioid carcinoma, NOS	184	70%
	Leiomyosarcoma	16	6.1%
	Mixed cell carcinoma*	1	0.4%
	Poorly differentiated carcinoma	1	0.4%
	Seromucinous carcinoma	1	0.4%
	Serous carcinoma	36	13.7%
	Undifferentiated carcinoma	2	0.8%

**Figure 1:**
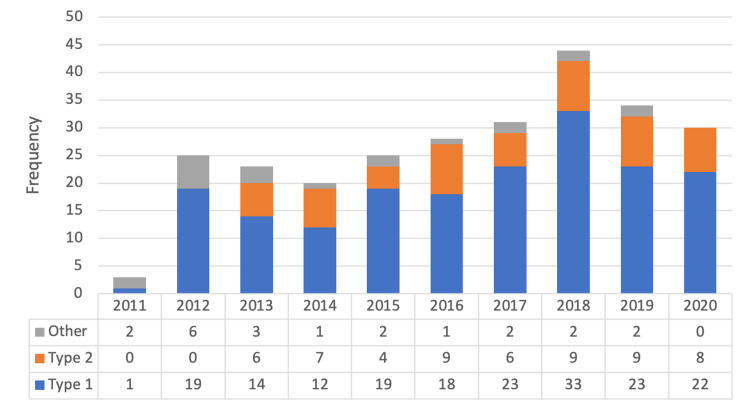
The distribution of histopathological diagnosis across years of study duration.

Histopathological diagnoses were classified into three types; Type 1, includes endometroid carcinoma, NOS; Type 2 includes carcinosarcoma, clear cell carcinoma, and serous carcinoma; Others included mixed cell carcinoma (serous carcinoma 90%, endometrioid carcinoma 10%), seromucinous carcinoma, leiomyosarcoma, poorly and undifferentiated carcinomas. Type one represented the majority with (70%), type 2 represented (22%), while others represented (8%) of the total. The types of histopathological diagnosis were demonstrated across the duration of years of the study. Other findings represented a minor proportion, while type 1 remained the most common diagnosis across the 10 years (Figure 1). Different management methods were used among the participants and the majority have had total abdominal hysterectomy (89.7%), while some have had dilation and curettage (4.6%), and other methods (6.1%). The details are shown in Table 2.

**Table 2:** Management of uterine malignancy among the patients

Therapeutic procedure	Yes		No	
	N	%	N	%
Total abdominal hysterectomy	236	89.7%	27	10.3%
Bilateral salpingo-oophorectomy	229	87.1%	34	12.9%
Debulking	30	11.4%	233	88.6%
Lymphadenectomy	49	18.6%	214	81.4%
Dilation and curettage	12	4.6%	251	95.4%
Omentectomy	17	6.5%	246	93.5%
Others	16	6.1%	247	93.9%

Histopathological diagnosis was tested with age, complaint, and radiological findings for statistically significant associations. While type 1 was the most common type among all the age groups, abdominal distention, and uterine masses showed a rise in proportion among patients with type 2 uterine malignancy (Table 3).

**Table 3:** The distribution of histopathological diagnosis across age groups, presenting complaints and radiological findings *Chi-square test; **Fisher exact test

Variables and groups	Diagnosis						
	Type 1		Type 2		Other		P-value
	N Total: 184	%	N Total: 58	%	N Total: 21	%	
Age							
30-40	16	61.5%	4	15.4%	6	23.1%	0.005*
41-50	23	63.9%	7	19.4%	6	16.7%	
51-60	55	79.7%	11	15.9%	3	4.3%	
>60	90	68.2%	36	27.3%	6	4.5%	
Complain							
Vaginal bleeding	160	76.6%	35	16.7%	14	6.7%	<0.001**
Abdominal pain	13	52.0%	9	36.0%	3	12.0%	
Abdominal distention	3	30.0%	5	50.0%	2	20.0%	
Others	8	42.1%	9	47.4%	2	10.5%	
Radiology							
No previous images	80	66.7%	30	25.0%	10	8.3%	<0.001**
Endometrial thickening	87	85.3%	13	12.7%	2	2.0%	
Uterine mass	15	53.6%	12	42.9%	1	3.6%	
Others	2	15.4%	3	23.1%	8	61.5%	

## Discussion

The most frequent malignancy reported of the female genital tract in developed and developing countries is uterine and cervical cancer, respectively [2]. In Saudi Arabia, uterus malignancy is the fourth most prevalent type of cancer among the female population proceeded by breast cancer, thyroid cancer, and colorectal cancer [2]. The frequency of the aforementioned tumors varies and is highly influenced by lifestyle choices, genetics, and socio-economic status [3,4]. A total of 263 uterine malignancy patients were analysed over 10 years from 2011 to 2020 with an age ranging from 30 to 95 years with a median age of 61 years.

The peak frequency of age at diagnosis in this study was significantly associated with age showing higher diagnoses among patients over 60 years of age representing 50.2% study population. Similar results were yielded from different studies showing the peak frequency among post-menopausal women which can be explained by the change of hormones in post-menopausal age [5,6]. Family planning was introduced in 1961 with contraceptive pills and condoms initially being used in 1970 and obtained full coverage in 1985 impacting the parity rate and increase of hormonal replacement therapy [6]. The frequency of structural uterine lesions increases in age such as polyps, atypical hyperplasia, and lesions on the ovary manifesting as women approach menopause [7]. This directly correlates to this study's findings which show older women of >60 years and above bearing over 50% of the uterine malignancy reported.

This study described endometrioid carcinoma, serous carcinoma, carcinosarcoma, and leiomyosarcoma in 70%, 13.7%, 7.2%, and 6.1% of the cases, respectively. As reported by Tanvir et al., a total of 38.8% of the cases had reports of endometrial thickening by radiology. The high reporting of endometrioid carcinoma could be attributed to the over diagnosis of hyperplasia, background polyps, endometritis, artifacts, and normal endometrium [8]. This stressed the combination of morphology and gross evaluation of the specimen, clinical history and examination, and appropriate sampling allowing correct diagnosis. The classical complaint correlated with endometrial cancer according to this study included abnormal vaginal bleeding affecting 79.5% of the patients, abdominal pain affecting 9.5%, and abdominal distention with 3.8%. The complaint significantly associated with endometrial cancer was abnormal vaginal bleeding.

Abnormal uterine bleeding (AUB) usually peaks in the above the 4th decade of the women as the physiological phenomenon of menopausal transition takes place. Risk assessment of women with AUB should be thoroughly determined with gynaecological evaluation [9]. Although in most cases of AUB, it is not linked to malignant or premalignant lesions, AUB should not be ignored. With the demographics of this study, AUB was highly reported in the >60 years population which was significantly reported for type 1 diagnosis and endometrial hyperplasia. In postmenopausal women with AUB, there is a risk of endometrial cancer of 10%, thus, the findings go in accordance with the literature available [10]. Other complaints were reported such as abdominal pain and distention by <10% of the study population.

The most frequent diagnosis in this study was endometrioid carcinoma with 70% of the study population categorizing as type 1 endometrial cancer. Type 1 endometrial cancer is more prevalent and reported diagnoses within an age group above 60 years. The most incidental cases of endometrial cancer were type 1 endometrioid carcinoma arising secondary to estrogen stimulation. Multiple studies had similar reports of higher type 1 cancer typically associated with obese, postmenopausal women contributed by hormonal stimulation [11]. High prevalence of type 1 endometrial cancer is observed throughout the decade compared to type 2 endometrial cancer which correlated with the findings of similar studies in both developing and developed countries [12]. 

The low prevalence of type 2 endometrioid carcinoma was associated with a short duration of symptoms, the absence of abnormal menstrual and generative functions, lack of signs of hyperoestrogenia and the absence of fat and carbohydrate metabolic disturbance. These factors such as lack of symptoms and signs conceal the malignancy leading to fewer diagnosed cases [13,14]. Similar findings were reported in studies describing the frequency of the histopathological morphology in uterine cancer showing a low frequency of type 2 endometrioid carcinoma [10,15].

The frequency rates of uterine carcinoma were found to be slightly increased from <5% of cases in 2011 to 25% in 2015 to 30% in 2020. It is important to note that the exponential increase in the frequency reported could also be due to the limited data resources available in the earlier years. Likewise, a study that analysed the frequency of uterine cancer in Thailand projected the incidence to rise from 1.5 to 5.3 per 100,000 women years in 2016 to 8 per 100,000 women years in 2030 [5]. The study attributed the rise in uterine malignancy to increased body mass index (BMI) ≥25 kg/m^2^, diabetes mellitus, and hypertension. The authors also reported that the shift in cultural values also impacts the rise in uterine malignancy such as an increase in early menarche, average age of marriage with decreasing parity, and large use of hormones in post-menopausal women which is similarly reported in multiple studies, applicable with the results from this study [8,15].

Our study highlighted the frequency, histopathological diagnosis, and common complaints of uterine carcinoma. However, there were study limitations. The study did not report tissue microassay, biomarkers, and immunohistochemistry which would be beneficially categorizing the uterine carcinoma reported. This would provide a large prospect for future clinical, histopathological, and epidemiological data on uterine cancer and the increasing reported frequency of cancer.

## Conclusions

The current study shows a trend of an increase in the incidence of uterine cancer through the last decade with an age distribution showing higher frequency among those over 60 years old. The most frequently reported histopathological diagnosis of uterine cancer was endometrioid carcinoma followed by serous carcinoma.

The current study recommends further molecular tests particularly those related to type 1 endometrial carcinoma in Saudi Arabia. This should also prompt increasing awareness of symptoms and signs among healthcare professionals and the public, avoiding the delays in identification of the malignancy.
